# Failure of asnis iii 5.0 mm cannulated screw: a case report

**DOI:** 10.1186/1757-1626-3-9

**Published:** 2010-01-07

**Authors:** A Chen, C Willis-Owen, K Akhtar, S Kamineni

**Affiliations:** 1 Department of Trauma and Orthopaedics, Charing Cross Hospital, Fulham Palace Road, London, UK

## Abstract

We describe a case of an ASNIS III 5.0 mm partially threaded cannulated screw unthreading itself as it was being inserted during fracture fixation of a humerus. The majority of complications associated with cannulated screws involve breaking of the screw, as opposed to unthreading. We believe that the self tapping design of the screw, in combination with the cannulated design, creates a potential area of weakness when used on hard bone.

## Introduction

The ASNIS cannulated Screw has been widely used in orthopaedics, with a 20 year clinical history. Cannulated screws, combined with image intensifier technology, have been a major facilitator of minimally invasive orthopaedic surgery, allowing for percutaneous fixation without major surgical exposure.

**Figure 1:**
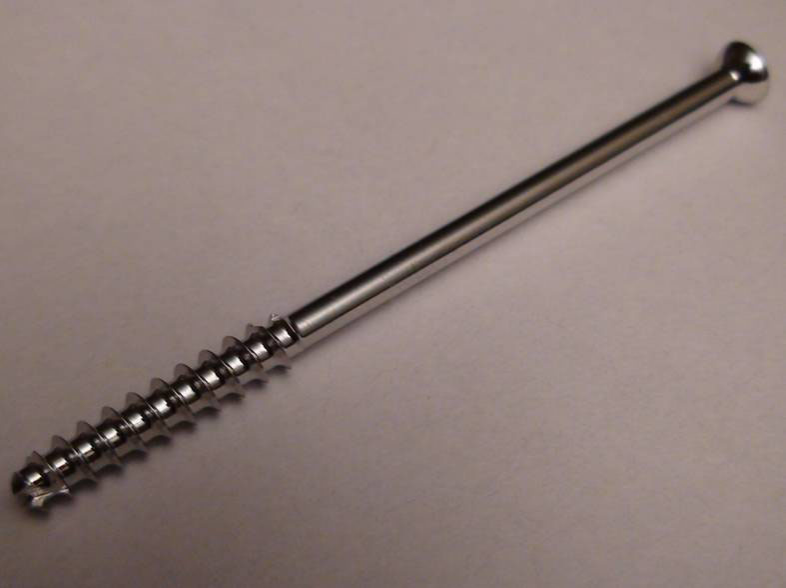
**Picture of Asnis III 5.0 mm Cannulated Screw**.

Use of cannulated screws, however, has not been without complications. The majority of these in the literature, however, involve breaking of the screw, either on removal or through instruments used such as drills and guidewires. In our search of the existing literature, there have been previous reports of cannulated screw failure during insertion - usually with the smaller diameter 4.0 mm AO SYNTHES screws. This case report documents a previously unrecorded failure of the ASNIS III 5.0 mm partially threaded cannulated screw system(Figure 1).

## Case presentation

A 30 year old Caucasian male presented to our hospital having fallen from a height and landing heavily on his left shoulder. On examination, he was tender over the proximal humerus and his left arm had no neurological deficits, with all pulses present. The gentleman sustained no other injuries. X-rays taken at the time of admission showed a 2-part fracture of the surgical neck of the left humerus, with rotational collapse of the humeral head. He was planned for surgery that weekend, on a trauma list supervised by the senior author, an upper limb specialist.

On the day of the surgery, a decision was made to attempt, in the first instance, percutaneous reduction of the fracture and fixation using cannulated screws. A small lateral stab incision was made under the fracture site, and a small periosteal elevator was used to raise the humeral head fragment. Provisional fixation was achieved using 3 crossing guidewires. Use of the image intensifier confirmed that the position was acceptable and the proximal cortex of the humerus was drilled and tapped over the guidewires. Two 5.0 mm cannulated screws were inserted from lateral to medial (one anterior, one posterior) without incident.

**Figure 2:**
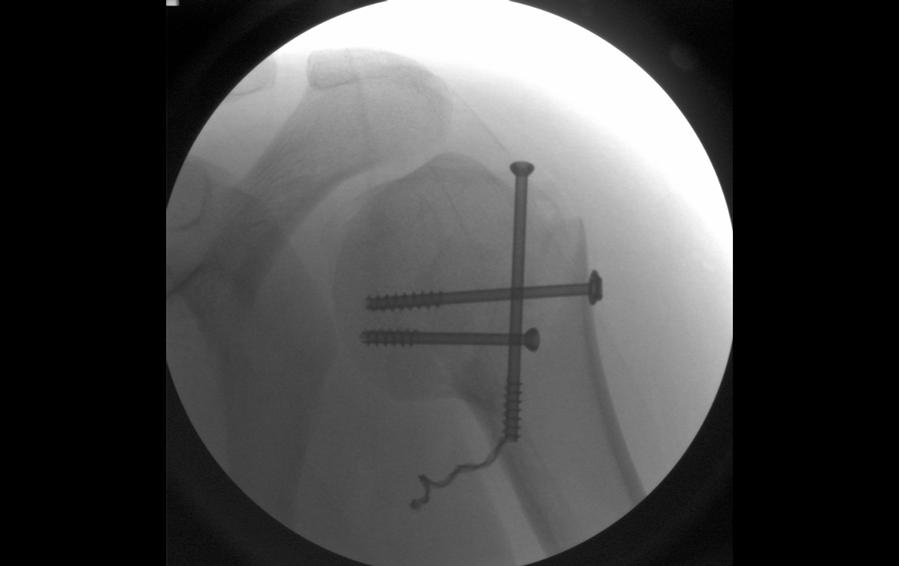
**Image of unthreaded screw at time of surgery**.

When the 5.0 mm superior-inferior cannulated screw was inserted, use of the image intensifier to confirm the final screw position revealed that the screw thread had unwound itself by 21/2 complete revolutions (Figure 2). There has no indication during manual insertion of the screw that this had occurred. Trying to reverse the screw out of the patient only succeeded in unthreading the screw further. While the unthreaded segment had not broken off, a decision was made not to attempt removal of the screw and the unthreaded segment to avoid causing any further damage to the neurovascular structures in the vicinity.

The gentleman was reviewed immediately post-recovery from the anaesthetic and found to have no neurovascular deficit in his left arm. The complication was explained to him and he was discharged the following day with a plan for weekly review for 3 weeks to check his neurovascular status.

**Figure 3:**
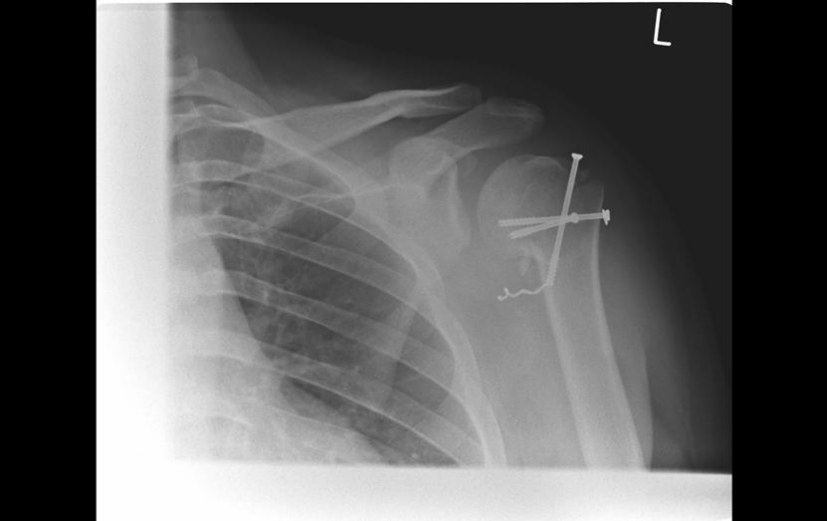
**Image of unthreaded screw 3 weeks following surgery**.

X-rays taken 3 weeks post-operatively(Figure 3) showed that the fracture had shifted slightly, but as the patient was comfortable and remained neurovascularly intact, a decision was made to gently mobilise the patient. Following discussion with the patient, it was decided not to attempt removal of the unthreaded screw.

## Discussion

Along with advancement in image intensifier technology, improved designs of cannulated screws have allowed orthopaedic surgeons to fix percutaneously many fractures that previously required open surgery. The lessened soft tissue trauma is believed to improve fracture healing.

There have been a number of complications noted with cannulated screw fixation. These included broken guidewires in the AO 3.5 mm system [1], and also many documentations of broken screws, including the AO 4.0 mm system [2-4]. There have been cases of screws unthreading in cancellous bone and one documentation of an AO 4.0 mm screw unthreading following contact with cortical bone, then breaking off in the elbow during cannulated screw fixation of a lateral epicondyle fracture [5]. All patients were young males and it has been hypothesized [2] and [5], that contact with dense bone caused the unravelling.

The exact aetiology of our screw failure is uncertain. In Levene's case [5], the surgeon experienced a tactile change informing him of the failure, but in all 3 of Mooney's cases [2], this did not occur. Likewise, in our case, there was no tactile feedback suggestive of the screw unthreading. By the time the fluoroscopic images were taken, a considerable length of screw thread had unwound.

We hypothesize that the self-cutting and self tapping fluted design of the screw, while facilitating ease of insertion, when combined with a cannulated screw design, may leave only a thin sliver of metal around the threads, which when rotated against dense bone whether cortical or cancellous has a potential to unravel.

The initial problems with the guidewires led to the development of thicker, stiffer wires to reduce the problems associated with wire breakage. However, increasing the diameter of the wires may have had a counterproductive effect of making the screws more susceptible to unravelling by thinning it.

It had previously been suggested [5] that pre-drilling and tapping and real-time fluoroscopy during screw insertion may be helpful in reducing the incidence of unravelling. In our case, the proximal cortex was pre-drilled and tapped prior to screw insertion. In addition, trying to reverse the screw out only succeeded in causing further unravelling. However, perhaps if the unthreading was noticed earlier using fluoroscopy, it may have been possible to remove the screw without incident.

## Conclusion

Orthopaedic surgeons need to be aware of this increasingly common complication with cannulated screw fixation, especially in young adults with dense bone. It should be noted that in the cases mentioned above, all patients were young men under the age of forty. Thus far, however, these complications have not had a significant adverse effect on the patients involved.

Mechanical testing of cannulated screws to confirm the torque necessary to unravel the threads may be a necessary addition the existing international strength and structural stability standards for orthopaedic implants.

## Consent

Written informed consent was obtained from the patient for publication of this case report and accompanying images. A copy of the written consent is available for review by the editor-in-chief of this journal.

## Competing interests

The authors declare that they have no competing interests.

## Authors' contributions

CWO was involved in the admission of the patient. SK carried out the surgery and AC was the major contributor in writing the manuscript. KA followed the patient up in clinic. All authors contributed to, read and approved the final manuscript.
